# Editorial: Bio and nanomaterials in tissue engineering and regenerative medicine (BioNTERM)

**DOI:** 10.3389/fbioe.2023.1193168

**Published:** 2023-04-04

**Authors:** Emilio Isaac Alarcon, Hasan Uludag, May Griffith, Diego Mantovani

**Affiliations:** ^1^ Department of Biochemistry, Microbiology, and Immunology, University of Ottawa, Ottawa, ON, Canada; ^2^ Department of Chemical & Materials Engineering, University of Alberta, Edmonton, AB, Canada; ^3^ Department of Ophthalmology and Institute of Biomedical Engineering, Montreal University, Montreal, QC, Canada; ^4^ Department of Mining, Metallurgical and Materials Engineering, Laval University, Quebec City, QC, Canada

**Keywords:** biomaterials, clinical translation, innovation, nanomateials, bioprinting

For many, 2020 will be remembered as the year our lives were put on hold. For scientists, the COVID-19 pandemic took away scientific conferences and student/faculty mobility programs, which are essential for diversifying ideas and establishing new collaborations. The field of biomaterials was no exception. With many groups and their students not being able to interact with peers, the editors of this unique Frontiers Research Topic, in collaboration with a talented team of trainees in Canada, decided to take matters into their own hands. This is how the virtual conference and workshop series on *Bio and nanomaterials in tissue engineering and regenerative medicine (BioNTERM)* was born. BioNTERM was first virtually held on March 2021 with plenary lectures and workshops. BioNTERM covered different but closely interrelated topics of bio and nanomaterials in tissue engineering ranging from Science Communication to 3D-bioprinting. With over 600 attendees from +20 countries, this free event was a tremendous success. The articles presented in this special Frontiers issue represent the diaspora of topics BioNTERM touched on.

The Research Topic of articles includes studies on translational materials such as a peptide-based material for on-the-spot cornea repair (Juarez et al.) and the impact of electron-bean irradiation on pre-made collagen-based corneal implants (Simpson et al.). Fundamental research on the effects of processing techniques for obtaining silk-fibroin (Wang Y et al.) and using plant viral nanoparticles as an additive for gelatin methacryloyl hydrogels for building complex 3D structures (González-Gamboa et al.). This Research Topic includes two mini-reviews that revise recent developments on using peptides to prepare biomaterials (Ross et al.) and some of the most common methodologies used in bioengineering lung scaffolds (Shakir et al.). Finally, a comprehensive review of the use of mRNA-containing biomaterials for bone repair is also part of this Research Topic (Wang J et al.).

The editors of this Research Topic would like to thank the scientists who contributed their work to this volume. While we are not still over the COVID-19 pandemic, we are convinced our scientific community has become stronger, more resilient, and highly connected. The lessons learned on using digital technologies to connect, network, and share scientific knowledge during the pandemic are crucial to building a more inclusive scientific and societal ecosystem for future generations.

